# The role of neurosurgery in medulla oblongata GBM, a challenge

**DOI:** 10.3171/2019.10.FocusVid.Editorial1

**Published:** 2019-10-01

**Authors:** Rudolf Fahlbusch

**Affiliations:** Department of Neurosurgery, International Neuroscience Institute, Hannover, Germany

A 43-year-old man with a short history, incomplete hemisymptomatic and cranial nerve IX and XII lesions, and an exophytic, unilateral (displaced pyramidal tract in DTI) ring-like medullary lesion shown on MRI.

Why not surgical exploration, if personal, even published, expertise in brainstem surgery and anatomical and neurophysiological knowledge about safe entry zones is practiced and documented convincingly in a video? The navigated resection of a circumscribed firmer tumor appears more like a lower-grade glioma than the finally diagnosed GBM—locally very rare in adults—avoiding additional neurological deficits. Intraoperative MRI could offer another potential for resection control.

It remains open how far “only biopsy” prior to tailored radio-chemotherapy—as a conventional procedure—would have changed the early and long-term outcomes.

